# Nuclear Molecular Imaging of Cardiac Remodeling after Myocardial Infarction

**DOI:** 10.3390/ph15020183

**Published:** 2022-01-31

**Authors:** Zohreh Varasteh, Wolfgang A. Weber, Christoph Rischpler

**Affiliations:** 1 Department of Nuclear Medicine, Klinikum Rechts der Isar der TUM, 81675 Munich, Germany; zohreh.varasteh@tum.de (Z.V.); w.weber@tum.de (W.A.W.); 2 Department of Nuclear Medicine, University of Duisburg-Essen, German Cancer Consortium (DKTK)—University Hospital Essen, 45147 Essen, Germany

**Keywords:** myocardial infarction, cardiac remodeling, inflammation, angiogenesis, fibrosis

## Abstract

The role of molecular imaging technologies in detecting, evaluating, and monitoring cardiovascular disease and their treatment is expanding rapidly. Gradually replacing the conventional anatomical or physiological approaches, molecular imaging strategies using biologically targeted markers provide unique insight into pathobiological processes at molecular and cellular levels and allow for cardiovascular disease evaluation and individualized therapy. This review paper will discuss currently available and developing molecular-based single-photon emission computed tomography (SPECT) and positron emission tomography (PET) imaging strategies to evaluate post-infarction cardiac remodeling. These approaches include potential targeted methods of evaluating critical biological processes, such as inflammation, angiogenesis, and scar formation.

## 1. Introduction

Because of advances in reperfusion therapy, acute mortality after myocardial infarction (MI) has decreased. However, the surviving patients are at increased risk of developing impaired infarct scar, adverse cardiac remodeling, and chronic heart failure.

The quality of intrinsic wound healing that occurs during the first 1–2 weeks after MI determines the fate of patients for years to come. Wound healing after cardiac ischemia is characterized by a first inflammatory phase in which the sterile wound recruits neutrophils and inflammatory leukocytes from the blood. Recruited monocytes differentiate into inflammatory macrophages that remove dead cells and extracellular matrix (ECM) debris. The inflammatory phase is then succeeded by more prolonged reparative and maturation phases of inflammation resolution and tissue repair that ultimately result in a stable scar after MI. Dysregulation in any of these distinct but overlapping stages in the wound healing process may cause adverse cardiac remodeling contributing to the pathogenesis of heart failure.

Although the exact predictors of the disease are not well-defined, the early detection, risk stratification, and prevention of adverse ventricular remodeling post-MI is the current focus of cardiovascular medicine. Molecular imaging approaches are emerging as the noninvasive detection of biological processes for diagnostic and prognostic purposes, which can differentiate affected tissues before the manifestation of gross anatomical modifications or physiological consequences. The success and increasing use of molecular imaging for the management of cardiovascular conditions rest in the development of high sensitivity nuclear imaging systems, that include single-photon emission computed tomography (SPECT) and positron emission tomography (PET), in combination with high spatial resolution CT or MRI, as well as novel targeted biological markers of molecular and physiological processes over the last two decades. Great interest lies in monitoring the time-related presence of all the phases mentioned above to predict cardiac wound healing after MI. In the following sections of this review, we will focus on the application of SPECT and PET molecular imaging in the evaluation of ischemic heart disease. These techniques include imaging of myocardial inflammation, angiogenesis, and fibrosis.

## 2. Inflammation

Following MI, monocytes, macrophages, and neutrophils invade the injured myocardium massively and constitute the initial inflammatory responses [1]. Optimal healing of the myocardium requires a suitable degree of acute inflammation. Insufficient or excessive acute inflammation is thought to impair myocardial salvage and promote chronic heart failure post-MI. In addition, timely resolution of inflammation is vital for optimal cardiac repair. Prolonged inflammation post-MI extends the infarction and promotes adverse cardiac remodeling. By modulating the activation of fibroblasts, inflammatory cells influence myocardial infarct healing and subsequent remodeling. Different experimental and clinical studies suggest that pharmacological interventions targeting inflammation following infarction may hold promise to reduce infarct size and prevent adverse cardiac remodeling and major adverse cardiac events [2]. Therapy with anti-inflammatory and immunomodulatory agents, however, may also inhibit important repair pathways, thus exerting detrimental actions in MI patients [1]. Therefore, identifying the therapeutic window for specific intervention is critical for an optimal therapeutic design in patients with MI. Molecular imaging of the inflammatory response may predict the individual risk for adverse outcomes and identify the optimal time window for therapeutic interventions.

Several studies have used different radiopharmaceuticals to target amino acid metabolism or specific receptors expressed by inflammatory leucocytes to delineate the extent and the severity of cardiac inflammation post-MI (Table 1).

**Figure 1:**
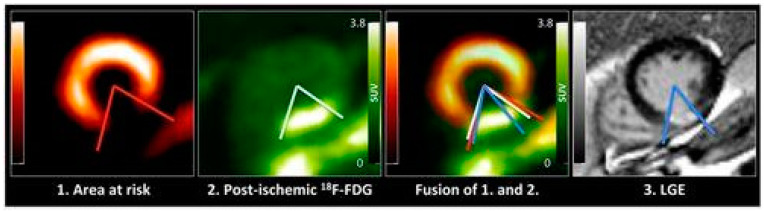
Increased myocardial FDG uptake in the postischemic myocardium reflects the inflammatory response. Early after myocardial infarction, there is an intense ^18^F-FDG uptake in the myocardium. The regional analysis of cardiac ^18^F-FDG and LGE extent confirmed that the ^18^F-FDG extent significantly exceeds the LGE extent. The area at risk depicted by ^99m^Tc-sestamibi SPECT matched roughly the post-ischemic ^18^F-FDG uptake [4].

Enhanced expression of glucose transporters and production of glycolytic enzymes in activated inflammatory cells is the most widely studied marker for imaging inflammation. In combination with late gadolinium enhancement (LGE) magnetic resonance imaging (MRI) or perfusion imaging, ^18^F-FDG PET has been historically applied to differentiate viable and non-viable cardiomyocytes in infarcted myocardium. However, recent preclinical studies have demonstrated that by adequately suppressing the physiological uptake of ^18^F-FDG in healthy cardiomyocytes, it is also feasible to utilize this technique to isolate the inflammatory signal and quantify monocyte/macrophage infiltration in the infarcted myocardium [3]. While the inhibition of physiological myocardial ^18^F-FDG uptake in animals was achieved with a specific anesthesia regimen (ketamine/xylazine versus isoflurane), specific measures such as dietary restrictions and/or heparin administration have to be followed in humans. Enhanced ^18^F-FDG uptake in the infarct zone compared to remote and healthy myocardium, in segments exhibiting LGE, and in segments with increased extracellular volume (ECV) fraction and native T1 relaxation time were observed in several clinical studies. Additionally, independent of infarct size, higher increased glucose metabolism and ^18^F-FDG uptake are associated with poorer left ventricular function (Figure 1) [4].

Upregulation of the G-protein-coupled receptor somatostatin receptor subtype-2 (SSTR-2) on the cell surface of activated macrophages was previously reported [5,6]. The low expression on unstimulated M0 macrophages and alternatively activated M2 macrophages and high expression on pro-inflammatory M1 macrophages make SSTR2 an ideal biomarker to image inflammation [6,7]. The concept of macrophage detection with SSTR-2 PET/CT compared to cardiac MRI in 6 male patients with sub-acute MI is investigated on a compassionate use base using ^68^Ga-DOTA-TOC [8]. Mild or moderate radiotracer retention was reported within areas of acute myocardial damage in 36 of 47 MRI positive segments (76.6%). No segment was rated with intense retention of the tracer. Similar findings have been reported for ^68^Ga-DOTA-TATE in another clinical study [9]. In a preclinical study, however, ^68^Ga-DOTA-TATE signal did not correspond to the regions of inflammation, suggesting that ^68^Ga-DOTA-TATE is not an appropriate tracer for evaluation of post-MI inflammation in mice [10]. High metabolic rate resulting in different tracer metabolism and clearance in mice compared to humans may be responsible for this discrepancy [11].

**Figure 2:**
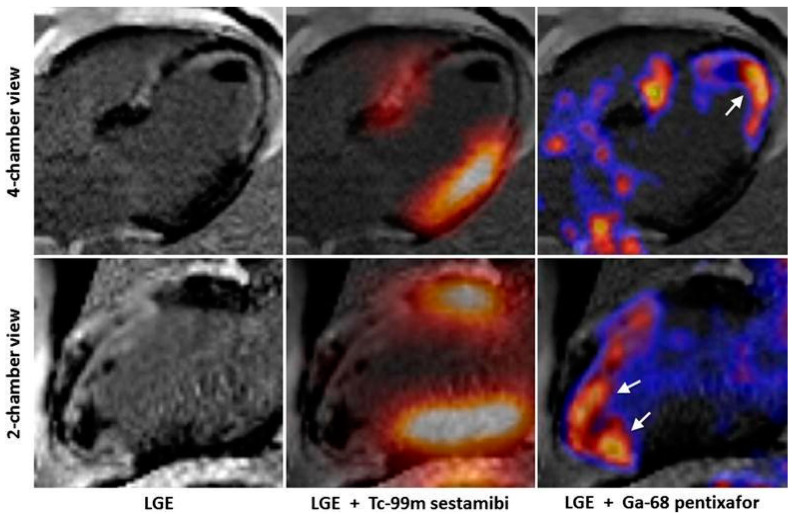
Overexpression of CXCR4 after myocardial infarction as a sign of inflammatory response. Early after myocardial infarction, the postischemic myocardium shows an intense accumulation of ^68^Ga-pentixafor, a radiotracer that binds to the chemokine receptor 4 (CXCR4). CXCR4 is overexpressed on inflammatory cells, such as activated macrophages [16].

The role of CXCR4, a transmembrane G-protein-coupled chemokine receptor, in cardiovascular disease has already been emphasized [12]. It was shown that CXCR4 and its cognate ligand CXCL12 play an important homeostatic function in leukocyte recruitment to the myocardium upon ischemic injury [12]. ^68^Ga-pentixafor as a molecular imaging marker of CXCR4 expression, which has primarily been used for imaging in tumors and lymphoproliferative disease [13,14,15], was tested for imaging inflammatory responses after MI (Figure 2) [16,17,18]. The feasibility of ^68^Ga-pentixafor PET imaging to visualize and quantify alterations in CXCR4 density has been assessed in mice and patients post-MI [17]. Pre-clinical data showed a consistently elevated uptake of ^68^Ga-pentixafor within the infarcted lesion peaking at 3 days post-MI. ^68^Ga-pentixafor PET imaging in patients, however, showed heterogeneous results indicating various degrees of CXCR4 upregulation and inflammatory responses in the infarct region [17]. Similar results with individual differences were reported in another clinical study [18]. Mild or moderate retention of ^68^Ga-pentixafor was reported in 3 of 7 patients [18]. Despite the presence of acute myocardial damage on CMR, 4 patients were ^68^Ga-pentixafor negative within 5 to 10 days after onset of clinical symptoms [18]. Although follow-up studies suggest that assessing CXCR4 expression after MI can reveal the myocardial healing potential [19], given the very limited number of observations, further studies are needed to assess if the extent of CXCR4-related inflammatory reaction and ^68^Ga-pentixafor uptake can predict heart failure after MI. ^68^Ga-pentixafor PET imaging might also enable proper patient selection for immunomodulatory therapy. Because of the significantly lower affinity of ^68^Ga-pentixafor for murine receptor, a mouse-specific PET tracer ^68^Ga-mCXCL12 was also investigated to assess the CXCR4 expression after initial acute MI in a mouse model of permanent left anterior descending coronary artery (LAD) occlusion. Compared to ^68^Ga-pentixafor, ^68^Ga-mCXCL12 showed more than 6 times higher mean %ID/g on day 3, indicating the higher specificity of ^68^Ga-mCXCL12 to the murine CXCR4 receptor. SPECT tracers have also been developed to this aim [20]. Radio-iodinated pentixather identified regional CXCR4 upregulation as early as 4 h after I/R in rat hearts. Li et al. showed that although the absolute ^131^I-pentixather uptake in the infarcted myocardium was slightly lower than that of ^68^Ga-pentixafor (1.9 vs. 2.2% ID/g), because of faster blood clearance (0.36% ID/g for ^131^I-pentixather vs. 2.0% ID/g for ^68^Ga-pentixafor, 30 min pi), ^131^I-pentixather demonstrated better imaging quality [20].

The recruitment of chemokine CC motif receptor 2 (CCR2)-positive (CCR2+) monocyte/macrophage subset to the injured myocardium was reported to stimulate pro-inflammatory responses, promote collateral tissue damage, and ultimately contribute to adverse remodeling and heart failure pathogenesis. ^68^Ga-DOTA-ECL1i, a radiotracer based on seven amino acid linear peptides with binding affinity to the allosteric position in CCR2, has been recently used to track the recruitment, accumulation, and resolution of CCR2+ leukocytes in two mouse models of sterile cardiac injury, including the reperfused MI model [21]. The significant uptake of ^68^Ga-DOTA-ECL1i localized to sites of tissue injury (mainly infarct and peri-infarct area) was associated with pro-inflammatory CCR2+ monocyte/macrophage content and abrogated in CCR2 deficient mice, demonstrating target specificity.

Further tracers of specific components of post-ischemic injury inflammation, including the amino acid ^11^C-methionine [22] as a marker of amino acid metabolism and protein turnover as well as several mitochondrial translocator protein (TSPO)-targeted radioligands [23] demarcated metabolically active immune cells and localized tissue inflammation in the injured myocardium.

Despite the progress in developing inflammation-targeted radiotracers, specificity for different inflammatory cell subpopulations is vaguely defined. The range of macrophage subpopulations extends far beyond the simplistic classifications of inflammatory M1 and anti-inflammatory M2 phenotypes, and their diverse (adverse or protective) roles highlights the need to design specific molecular-targeted radiotracers to achieve selectively targeting and clearly elucidating the specific subtypes and hence differentiating distinct phases of the inflammatory pathway after MI. In addition, molecularly specific imaging of the inflammatory process and isolating diverse cell populations can be used to identify therapeutic windows to inhibit the adverse effects of inflammatory macrophages and preserve the beneficial effects of anti-inflammatory subsets.

## 3. Angiogenesis

The natural healing process following MI involves a robust angiogenic response that commences in the ischemic border zone and extends toward the core region of the necrotic area. Triggered by hypoperfusion and tissue hypoxia, angiogenesis results in the formation of new capillaries from pre-existing microvessels and partial restoration of blood perfusion in the ischemic zone. The extent of angiogenesis has been reported to have positive implications for the prognosis of patients post-MI.

In the current MI patient care, the pro-angiogenic effect of novel therapies are assessed by altered myocardial blood flow using, e.g., ^201^Tl/^99m^Tc-sestamibi, ^13^NH_3_, or ^15^O-labelled water [24]. Although improved myocardial perfusion can be used as indirect evidence for angiogenesis in the infarcted myocardium and to indicate recovery, these techniques lack the sensitivity to monitor angiogenesis and predict the efficacy of pro-angiogenic treatment outcomes. It often takes several months before any improvement can be detected using perfusion imaging. To monitor the temporal progression of angiogenesis, to reliably assess the efficacy of pro-angiogenic therapies, and to improve treatment options, a sensitive and specific biomarker for direct imaging of angiogenesis is warranted. Attempts to directly visualize angiogenesis in cardiac regeneration after MI have been made using different radiotracers (Table 2).

Upregulation of different growth factors, including vascular endothelial growth factor (VEGF) post-MI, is well documented. VEGF as the most prominent member of a family of growth factors is strongly associated with angiogenic stimuli in different pathophysiologic situations, including left ventricular remodeling post-MI [25]. Previous studies have demonstrated an initial increase in the expression of VEGF and a subsequent increase in the expression of the VEGF receptors (VEGFRs) in the myocardium post-MI. Recombinant human VEGF_121_ was labeled with ^64^Cu (^64^Cu-DOTA-VEGF_121_) for serially monitoring VEGFR expression after MI [26]. ^64^Cu-DOTA-VEGF_121_ uptake peaked at three days post-MI and corresponded to VEGFR expression. Fused PET images of ^18^F-FDG and ^64^Cu-DOTA-VEGF_121_ showed that the ^64^Cu-DOTA-VEGF_121_ myocardial signal matched well with the areas of infarcted myocardium, as evidenced by the absence of ^18^F-FDG uptake.

Integrins are heterodimeric transmembrane glycoproteins consisting of 1 of 18 α-chains and 1 of 8 β-subunits, which regulate cell-cell and cell-extracellular matrix (ECM) interactions. Several members of this structurally and functionally diverse family are overexpressed on endothelial cells (ECs) under hypoxia [27]. Several peptides containing the tripeptide Arg-Gly-Asp (RGD) sequence with high affinity to the α_v_β_3_ and α_v_β_5_ integrins, which become highly expressed in activated ECs during angiogenesis, have been applied for in vivo imaging of angiogenesis in the infarcted myocardium [28,29,30]. Some tracers were also utilized to assess the effect of treatment on myocardial angiogenesis post-MI [31]. ^99m^Tc-NC100692 is being developed as a marker of vitronectin integrin receptors that are associated with angiogenic endothelium [32]. With a high affinity to α_v_β_3_ and α_v_β_5_ integrins, ^99m^Tc-NC100692 has been recently utilized to assess the efficacy of neprilysin inhibitor sacubitril and angiotensin receptor blocker valsartan (SAC/VAL) therapy on myocardial remodeling and cardiac perfusion in a rat model of heart failure following MI [31]. An increase in the uptake of ^99m^Tc-NC100692 within infarct territory was observed in treated groups compared to the controls. Although in vivo mapping of α_v_β_3_ and α_v_β_5_ integrins being thoroughly investigated in recent years, their clinical value is still not well defined. 

Genetic studies in mouse models indicate that neither α_v_ nor β_3_/β_5_ integrins are essential for vascular development and ablation of the gene for the α_v_, β_3_, or β_5_ integrin subunits can be compensated by upregulation of other pathways, such as VEGF receptor-2 expression [33,34,35]. Additionally, integrin α_v_β_3_ expression is not restricted to ECs, but it is also frequently expressed on macrophages and activated fibroblasts [36,37]. Therefore, the results in angiogenesis imaging targeting integrins α_v_β_3_ or α_v_β_5_ should be treated carefully. In contrast to the observations made for α_v_β_3_ and α_v_β_5_ integrins [33,34], α_5_β_1_ expression was reported to be completely restricted to angiogenic ECs, suggesting that integrin α_5_β_1_ could be a more reliable biomarker for imaging angiogenesis. A PET radiotracer based on α_5_β_1_ integrin-targeted trimeric pseudopeptide, ^68^Ga-aquibeprin, was already developed and evaluated in tumor xenografted mice [38].

Endoglin (CD105) is a 180 kDa disulfide-linked homodimeric transmembrane protein that is selectively expressed on the ECs of newly formed vessels [39]. A radiotracer based on a chimeric monoclonal antibody that binds to both human and murine CD105 has been developed and evaluated for non-invasive imaging of CD105 expression in a rat model of MI [40]. However, the long circulation half-life of the radiolabeled antibody, which resulted in an intense background signal, acted as a confounder.

Upregulation of CD13 on activated ECs of angiogenic vessels and the homing of a cyclic tripeptide Asn-Gly-Arg (cNGR) to CD13 expression in the infarcted area and border zones of the myocardium was reported earlier [41,42]. Following fluorescently labeled cNGR conjugates as well as cNGR-labelled paramagnetic quantum dots for fluorescence and MR imaging, respectively, an indium-111-labeled SPECT probe based on cNGR, ^111^In-DTPA-cNGR, was developed for sensitive molecular imaging of angiogenesis [43]. Target-specific uptake of the tracer in areas of decreased perfusion was revealed by analysis of acquired SPECT images in the AHA 17 segment model [43]. 

A comprehensive summary of preclinical and clinical studies on noninvasive in vivo molecular imaging of angiogenesis in cardiac regeneration after MI has been provided in a review by Mandic et al. [29].

## 4. Fibrosis and Fibrosis Activity

Cardiac fibrosis is the formation or development of excess fibrous connective tissue in the myocardium as a result of a reparative or reactive process. Following MI, necrotic myocytes are replaced by ECM components (largely type I collagen) to form a fibrotic myocardial scar. Initially formed fibrotic tissue protects the heart from rupture. However, as MI evolves to the late stages, the fibrotic tissue expands to non-infarcted remote myocardium, leading to a progressive increase in ventricular stiffness and a decrease in myocardial contractility. The heightened cardiac fibrosis is implicated in adverse outcomes and reported as an independent predictor of worse long-term survival [44]. Therefore, cardiac fibrosis has been recognized as a target for post-MI therapeutics. Non-invasive imaging modalities are necessary for the accurate assessment of cardiac fibrosis. Cardiac MR (CMR) has traditionally been used to diagnose and follow-up post-MI fibrosis. Myocardial fibrosis can be imaged using two different MR techniques. LGE method detects areas of macroscopic replacement fibrosis, while T1 mapping images diffuse and microscopic interstitial fibrosis. Besides clinically established MR techniques, molecular imaging modalities are likely to add the understanding of pathophysiological mechanisms and insight into the rate of disease progression or regression (in response to therapy).

The collagen-targeted MR contrast agents have been tested as the first specific probs for molecular imaging of post-MI fibrosis [45,46]. Radiolabeled probs targeting collagen have also been introduced for sensitive, specific, and quantitative detection of fibrosis in multiple murine studies [47,48]. ^99m^Tc-streptavidin-coupled-collagelin, a peptide-based probe with collagen-binding properties, has been tested for pathological accumulation of collagen in a rat model of healed MI [47]. The significant uptake of ^99m^Tc-streptavidin-collagelin in the MI scar of all rats was reported. In contrast, no tracer uptake was observed in the myocardial tissue of sham-operated rats or MI rats receiving ^99m^Tc-labeled control peptide [47]. ^99m^Tc-CBP1495, another collagen-targeted peptide tracer with relatively high affinity to collagen type I, was utilized for quantitative evaluation of tissue fibrosis. SPECT images revealed significantly enhanced uptake of ^99m^Tc-CBP1495 in fibrotic tissue of pulmonary and hepatic fibrosis rat models [48]. An overview of ECM imaging, including the molecular targeting of key players involved in ECM deposition and degradation (e.g., matrix metalloproteinases), has been extensively reviewed by De Haas et al. [49]. It is important to note that ECM remodeling is a relatively late pathogenetic process in post-MI heart failure progression, which may limit the benefit for the patients.

Fibrosis is the consequence of fibroblast activation. The methodologies imaging fibrosis tend to reflect a relatively late product of fibroblast activation and are less sensitive at detecting the early stage of the disease. Additionally, the imaging techniques targeting altered ECM composition detect established areas of diffuse and replacement myocardial fibrosis and cannot distinguish active ongoing fibrosis. Molecular targeting of activated (myo)-fibroblasts, however, can depict the active disease at a very early stage where treatments would likely have the most benefit. Imaging of myocardial angiotensin II (AT) type-1 receptor (AT1R) as a target for visualization of α-smooth muscle actin-positive collagen-producing myofibroblasts has been evaluated in different experimental MI models. SPECT imaging with ^99m^Tc-losartan in murine [50] and PET imaging with ^11^C-KR31173 in porcine [51] MI models revealed that assessing myocardial AT1R expression is feasible using non-invasive nuclear imaging techniques. Early identification of proliferating myofibroblasts and measurements of fibrosis activity was pursued by targeting the α_v_β_3_ integrin, using RGD imaging peptides in post-infarct animal models and patients [28,30,52,53,54]. ^99m^Tc-Cy5.5-RGD (CRIP) was also used to evaluate the therapeutic effects of anti-angiotensin and anti-aldosterone neurohumoral antagonists on interstitial alterations, individually or in combination after MI in mice [54]. ^99m^Tc-CRIP uptake correlated with the extent of thin collagen fibers deposition and reconfirmed superiority of combination therapy. However, as mentioned earlier, preclinical and clinical studies documented that upregulated expression of α_v_β_3_ is not restricted to activated cardiac myofibroblasts, but it is also frequently expressed on macrophages [36] and within activated endothelial cells (ECs) of the microvasculature [55,56]. Therefore, even though α_v_β_3_ expression may hold promise as a combined biomarker of postinfarct healing activity (inflammation, fibroblast activation, and angiogenesis), it is not exclusively specific for activated fibroblasts [57]. Accordingly, more specific biomarkers of fibroblast activity have been desirable.

**Figure 3:**
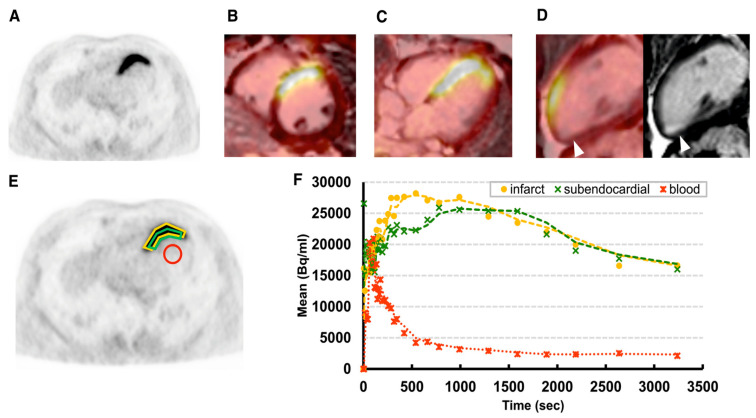
Imaging fibroblast activity after myocardial infarction. Early after myocardial infarction, fibroblast activation occurs. This can be visualized in PET using the tracer ^68^Ga-FAPI (fibroblast activation protein inhibitor). In the example shown, intense fibroblast activation can be observed in the infarct area of the septal and anteroseptal region (**A**–**F**). On MRI (**D**), a small area of mature scar most likely stemming from a prior infarction can be seen. On PET, no increased ^68^Ga-FAPI uptake is seen in this mature scar [60].

The serine protease fibroblast activation protein (FAP) is a transmembrane-anchored enzyme with a restricted expression pattern in fibroblasts that are activated to differentiate to (proto-)myofibroblasts. It is not expressed in dormant fibroblasts or mature fibrocytes [58]. Therefore, imaging of FAP expression allows one to study fibroblast activity early after activation of the fibroblasts. FAP inhibitor (FAPI) tracers were initially developed as theranostic ligands for metastasized cancer. Recently, they have been used to study cardiac remodeling following MI in small-animal models [59] and in humans (Figure 3) [60,61]. The tracers were also used to investigate cardiac remodeling in patients who underwent FAPI PET imaging for cancer staging [62,63,64], in patients with a checkpoint inhibitor (ICI)-associated myocarditis [65], with nonischemic dilated cardiomyopathy [66], and to image cardiotoxicity in patients after receiving systemic cancer therapy [67]. Recently, another FAP-targeting PET radiotracer, ^68^Ga-MHLL1, was evaluated for non-invasive tracking of dynamic fibrosis in MI mice [68]. A single-step synthesis and semi-preparative HPLC purification of the tracer resulted in moderate radiochemical purity with two major impurities, which resulted in unspecific uptake in the liver and gallbladder. ^68^Ga-MHLL1 displayed selective binding to FAP in the infarct and infarct border zone, as well as remote non-infarcted myocardium, post-MI. Dedicated prospective clinical studies will be necessary to determine the added value of FAP imaging for predicting functional outcomes in cardiac patients. The development of FAP-targeted radiopharmaceuticals and their application in nuclear medicine has been recently reviewed by Lindner et al. [69]. Different radiopharmaceuticals targeting activated fibroblasts/myofibroblasts as well as their extracellular matrix products post-MI are listed in Table 3.

Similar to leukocytes/macrophages, fibroblasts exhibit extensive functional heterogeneity. Therefore, specific targeting of fibroblast subtypes and isolating diverse cell populations with adverse or reparative functions can provide critical insights into disease progression and response to therapy. 

## 5. Cardiac Molecular Imaging in Therapeutic Applications

The traditional surgical or drug treatments after MI, including coronary angioplasty, coronary artery bypass graft, and thrombolytic therapies, can effectively reduce the size of infarction by saving the dying cardiomyocytes and delaying left ventricular remodeling. However, they cannot modify the local tissue environment and support intrinsic repair. In addition, the extent of adverse cardiac remodeling after MI is only partially dependent on the infarct size. Some patients with relatively small infarcts develop adverse ventricular remodeling, while others with larger MIs do not. The excess of cardiac remodeling is also very much affected by the quality of cardiac wound healing and the cellular and molecular profile of the viable myocardium. The comprehensive reviews on advances in the development of immune-modulating therapeutic interventions targeting specific inflammatory pathways following MI, novel strategies to promote therapeutic angiogenesis in the infarcted area, and systemic or localized delivery of anti-fibrotics, including the approaches to inhibit myofibroblast formation that favorably influences wound healing and repair after MI have been published recently [1,70,71,72,73]. Due to mainly pathophysiological heterogeneity in human patients, however, clinical validation of these novel strategies faces a challenge. Molecular imaging probes can identify patient-specific pathophysiologic makeup, stratify patient populations, and select potential responders to include in clinical trials. Molecular imaging techniques can also be used to monitor therapeutic efficacy at earlier time points soon after treatment initiation, which is not achievable with conventional anatomical or physiological methods. Altogether, molecular imaging strategies are likely to greatly accelerate the translation of novel therapeutics for post-infarction cardiac repair.

Several novel radionuclide image-guided molecular repair concepts are emerging in cardiology. Recently, an image-guided early CXCR4 directed therapy was tested in mouse MI models [74]. Hess et al. could clearly demonstrate the dynamic changes of cardiac CXCR4 upregulation post-MI using ^68^Ga-pentixafor˗PET. They showed that CXCR4 inhibition (by AMD3100) at the time of maximum PET signal (day 3) accelerates inflammatory resolution and improves cardiac wound healing. However, the off-peak (day 7) CXCR4 blockade did not improve the cardiac outcome in MI mice, highlighting the importance of optimal timing for initiation of the therapy to maximize treatment efficacy [74]. Specific radiotracers holding potential for image-based guidance of target and timing of cardiac reparative interventions post-MI are summarized by Hess et al. [73].

Recently, the field of cardio-oncology has been recognized as an integral part of precision oncology that focuses on the identification, prevention, monitoring, and treatment of cardiovascular events occurring as side effects of cancer treatments. Many cancer patients have pre-existing risk factors or cardiovascular diseases, which can lead to a broad spectrum of cardiovascular complications because of the cancer itself and/or, in particular, cancer therapies. Many cancer patients undergo several oncological therapies, which any of those therapies should be considered an independent risk factor for a future potentially cardiotoxic event, especially in patients with previous cardiovascular events like MI. This consideration is particularly true for the anthracyclines that are extensively used to treat acute leukemias, breast carcinomas, sarcomas, and malignant lymphomas. Anthracycline class (including doxorubicin, epirubicin, daunorubicin, idarubicin, mitoxantrone) is associated with a considerable risk of myocardial damage, particularly at high doses [75]. While cardiovascular toxicity associated with anthracyclines is well defined, the evidence for targeted agents is still emerging. Because of shared cardiovascular protein signaling pathways, many targeted therapies for cancer have been associated with cardiovascular toxicity. It is very crucial to identify patients at high risk for therapy-associated cardiotoxicity prior to any oncological therapy. In this regard, molecular imaging can be used not only for the diagnosis and staging of cancer but also for early detection of pre-existing cardiac disease before cancer therapy and to monitor patients for early detection of cardiac alterations during cancer therapy. The major types of cardiotoxicities associated with frequently used chemotherapeutic agents and the role of cardiac molecular imaging in assessing cancer therapeutics-related cardiac dysfunction have been reviewed by Sreenivasan et al. [75]. Cardiac FAPI uptake as a sign of cardiotoxicity at its early and functional stage was reported recently in a patient with pancreatic ductal adenocarcinoma receiving systemic cancer therapy [67]. The feasibility of monitoring immune checkpoint inhibitors (ICIs)-induced inflammatory and fibrotic reactions in the heart have been evaluated using ^68^Ga-DOTA-TATE and ^68^Ga-FAPI, respectively [65,76].

## 6. Conclusions

Molecular imaging is quickly being recognized as a tool meeting the increasing demands associated with precision medicine by studying not only the disease process but, more importantly, the efficacy of an individually tailored therapeutic regimen. Also, after MI, to track both disease and therapeutic intervention, reliable and sensitive non-invasive imaging tools are needed to assess the state of the target molecule before and after any intervention is carried out. In contrast to well-established PET and SPECT radiotracers in clinical oncology, there are still many limitations for developing radiopharmaceuticals for cardiac diseases. However, it is evident that as therapeutic approaches are evolving, imaging approaches to measure cardiac repair must also adapt. Molecular imaging of inflammation, angiogenesis, and fibrosis/fibrosis activity bear the potential to guide new therapies aiming for optimal wound healing following MI. All the advancements in developing novel radiopharmaceuticals for specific targets in cardiovascular imaging confirm that the revolution in personalized medicine is just at its beginning.

## Figures and Tables

**Table 1:** Radiopharmaceuticals for molecular imaging of post-MI inflammation.

Marker	Probe	Imaging Modality	Application
Glucose transporters (GLUTs)	^18^F-FDG	PET	Preclinical/clinical
SSRT-2	^68^Ga-DOTA-TOC	PET	Preclinical/clinical
	^68^Ga-DOTA-TATE	PET	Preclinical/clinical
Chemokine receptors (CXCR4, CCR2)	^68^Ga-pentixafor	PET	Preclinical/clinical
	^131^I-pentixather	SPECT	Preclinical
	^68^Ga-DOTA-ECL1i	PET	Preclinical
Mitochondrial membrane translocator protein (TSPO)	^18^F-GE180	PET	Preclinical
Amino acid metabolism and protein turnover	^11^C-methionine	PET	Preclinical/clinical

**Table 2:** Radiopharmaceuticals for molecular imaging of post-MI angiogenesis.

Marker	Probe	Imaging Modality	Application
VEGF	^64^Cu-DOTA-VEGF_121_	PET	Preclinical (rat MI model)
α_v_β_3/_α_v_β_5_ integrins	^18^F-galacto-RGD ^68^Ga-NODAGA-RGD ^68^Ga-TRAP-(RGD)_3_ ^68^Ga-NOTA-RGD ^18^F-Alfatide II ^68^Ga-RGD ^99m^Tc-RAFT-RGD ^68^Ga-PRGD2 ^18^F-Fluciclatide ^99m^Tc-NC100692	PET PET PET PET PET PET SPECT PET PET SPECT	Preclinical (rat MI model) Preclinical (rat MI model) Preclinical (rat MI model) Preclinical (rat MI model) Preclinical (rat MI model) Preclinical (rat MI model) Preclinical (rat MI model) Clinical Clinical Preclinical (rat model of MI)
CD105	^64^Cu-NOTA-TRC105	PET	Preclinical (rat model of MI)
CD13	^111^In-DTPA-cNGR	SPECT	Preclinical (Mouse MI model)

**Table 3:** Radiopharmaceuticals for molecular imaging of post-MI fibrosis and fibrosis activity.

Marker	Probe	Imaging Modality	Application
Fibrosis collagen	^99m^Tc-streptavidin-B-collagelin	SPECT	Preclinical (rat MI model)
Fibroblast activity Angiotensin receptor	^99m^Tc-Losartan	SPECT	Preclinical (mouse MI model)
	^11^C-KR31173	PET	Preclinical (pig MI model)/clinical
Fibroblast activity α_v_β_3_ integrin	^18^F-galacto-RGD ^99m^Tc-Cy5.5-RGD (CRIP) ^99m^Tc-RGD (RIP)	PET SPECT SPECT	Preclinical (mouse and rat MI model)/clinical Preclinical (mouse MI model) Clinical
Fibroblast activity FAP	^68^Ga-FAPI-04	PET	Preclinical (rat MI model)/clinical
	^68^Ga-FAPI-46	PET	Clinical
	^68^Ga-MHLL1	PET	Preclinical (Mouse MI model)

## Data Availability

Data sharing is not applicable.
